# The Petri-Dish Effect

**DOI:** 10.1017/dmp.2020.67

**Published:** 2020-04-03

**Authors:** Oluwafunbi Awoniyi

**Affiliations:** Department of Emergency Medicine, Beth Israel Deaconess Medical Center, Boston, MA

**Keywords:** Coronavirus, Diamond Princess cruise ship, quarantine

COVID-19 is caused by the virus SARS-CoV-2, a member of the coronavirus family known for causing varieties of the common cold, but also more serious diseases, as occurred with the SARS and MERS outbreaks. COVID-19 was first detected in Wuhan City, China, and linked to a live animal market. According to the World Health Organization, “the virus which causes COVID-19 most probably has its ecological reservoir in bats, and transmission of the virus to humans has likely occurred through an intermediate animal host – a domestic animal, a wild animal or a domesticated wild animal which has not yet been identified.”^1^ The virus currently is spreading from person-to-person via close contact, respiratory droplets, or surface contact. On February 3, 2020, the Diamond Princess cruise ship docked in Yokohama, Japan, with 3600 passengers. The passengers on the ship were quarantined due to 10 initial confirmed cases of COVID-19.^2^ While patients with confirmed cases were taken off the ship for treatment, other passengers were quarantined for 14 days (the scientific community’s consensus incubation period) in their rooms on the ship. As of February 25, 2020, 542 passengers had been confirmed with the virus. While passengers were being taken off the ship, the number of passengers infected continued to increase.

Any infectious disease outbreak can be broken up into 5 stages: detection, isolation containment, verification, treatment, and eradication. The containment stage is when society has the opportunity to contain the spread of disease and mitigate its progression to an epidemic or pandemic. If containment fails, the disease can spread and, depending on the transmissibility (R0) and death rate of the disease, the results can be catastrophic. On the other hand, a poorly executed quarantine process within the area of containment can also allow further spread of the disease among those being quarantined. Thus, it is important to critically assess the containment and quarantine strategies used on cruise ships during the current COVID-19 outbreak. Quarantine procedures included asking passengers to stay in their rooms, wearing masks, and walking on the deck for only several minutes each day. Some have praised cruise ship containment as being effective in reducing the spread to the mainland, but public health experts have criticized this process as risky to passengers and believe that the ship could create a Petri-dish effect. This could increase the number of confirmed cases among passengers and crew who worked and slept in close quarters.^3,4^ According to the President of World Association for Disaster and Emergency Medicine, Dr Gregory Ciottone, “These cruise ship quarantines as currently being implemented should change. Due to the sequential exposure/infection rate (ventilation system) there is no definitive end-time, and all onboard will only be disembarked either after infected, or when they may be in an asymptomatic incubation period.”^5^

Methods to improve quarantine on cruise ships include dividing the cruise ship passengers into 3 groups. The first group of symptomatic patients who test positive for the virus would be taken off the ship and be transported to a medical facility where they can be placed under strict isolation and undergo treatment. This isolation may include negative pressure rooms for patients and the necessary PPE for healthcare workers involved in their care, such as, N-95 masks, gloves, and gowns. Remaining passengers on the ship who are not symptomatic should be tested for the virus to determine who should go in the second and third groups. Those who test positive but are asymptomatic should be taken off the ship and placed in a temporary medical tent. This would need to contain isolation rooms for each passenger where they would be monitored for progression or regression of their condition. The third group would consist of those who test negative. This group would be under strict quarantine for 14 days, as they may have been exposed to the virus, to observe for signs of developing infection. Placing the passengers into these 3 groups would help reduce the spread of the virus from passengers in group 2 to those in group 3, and thus ensure that infected passengers get the care they need without forcing others to be continually exposed.

Like cruise ships, apartment buildings are full of people who live in close proximity with others and use common areas, and this may make for a comparable breeding ground for the Petri-dish effect to occur. High rise apartment buildings have multitudes of people interacting every day, whether in elevators, common rooms or the laundry. As of April 1, 2020, COVID-19 has been confirmed in 47,439 cases in New York City alone with 1,941 deaths.^6^ Apartment complexes in New York and other large cities must take precautionary measures to keep tenants safe, such as social distancing strategies, placing hand sanitizers in all public spaces, frequent cleaning of elevators, closing recreational rooms, and providing alcohol wipes in the laundry areas. This will help limit the spread of COVID-19, and also encourage tenants to continue proper hand hygiene in their own apartments.
